# When to stop social learning from a predecessor in an information-foraging task

**DOI:** 10.1017/ehs.2024.29

**Published:** 2025-01-20

**Authors:** Hidezo Suganuma, Aoi Naito, Kentaro Katahira, Tatsuya Kameda

**Affiliations:** 1 Department of Social Psychology, The University of Tokyo, 7-3-1 Hongo, Bunkyo-ku, Tokyo 113-0033, Japan; 2 School of Environmental Society, Institute of Science Tokyo, 3-3-6 Shibaura, Minato-ku, Tokyo 108-0023, Japan; 3 Japan Society for the Promotion of Science, 5-3-1 Kojimachi, Chiyoda-ku, Tokyo 102-0083, Japan; 4 Human Informatics and Interaction Research Institute, National Institute of Advanced Industrial Science and Technology, Tsukuba, Ibaraki 305-8566, Japan; 5 Faculty of Mathematical Informatics, Meiji Gakuin University, 1518 Kamikuratachou, Totsuka-ku, Yokohama, 244-8539 Japan; 6 Center for Interdisciplinary Informatics, Meiji Gakuin University, 1-2-37 Shirokanedai, Minato-ku, Tokyo 108-8636, Japan; 7 Center for Experimental Research in Social Sciences, Hokkaido University, N10W7, Kita-ku, Sapporo, Hokkaido 060-0810, Japan; 8 Brain Science Institute, Tamagawa University, 6-1-1 Tamagawagakuen, Machida, Tokyo, 194-8610 Japan

**Keywords:** social learning, exploration–exploitation trade-off, information foraging, intergenerational transmission

## Abstract

Striking a balance between individual and social learning is one of the key capabilities that support adaptation under uncertainty. Although intergenerational transmission of information is ubiquitous, little is known about when and how newcomers switch from learning loyally from preceding models to exploring independently. Using a behavioural experiment, we investigated how social information available from a preceding demonstrator affects the timing of becoming independent and individual performance thereafter. Participants worked on a 30-armed bandit task for 100 trials. For the first 15 trials, participants simply observed the choices of a demonstrator who had accumulated more knowledge about the environment and passively received rewards from the demonstrator's choices. Thereafter, participants could switch to making independent choices at any time. We had three conditions differing in the social information available from the demonstrator: choice only, reward only or both. Results showed that both participants’ strategies about when to stop observational learning and their behavioural patterns after independence depended on the available social information. Participants generally failed to make the best use of previously observed social information in their subsequent independent choices, suggesting the importance of direct communication beyond passive observation for better intergenerational transmission under uncertainty. Implications for cultural evolution are discussed.

## Graphical abstract

**Figure:**
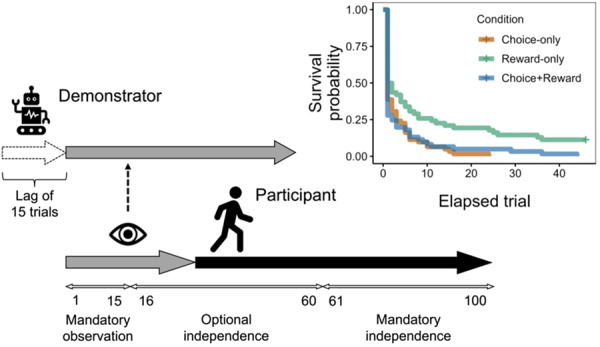

**Social media summary:** Humans strategically learn from predecessors under uncertainty, depending on the types of information available.

## 1. Introduction

Social learning is a key element of human intelligence. As implied by the phrase ‘standing on the shoulders of giants’ in scientific research (Merton, 1965), our intelligence is augmented by inheriting knowledge from our predecessors and elaborating on it with contemporaries. At the level of individual development, children initially receive a great deal of cultural information from their parents and close adults (vertical/oblique transmission), but as they grow up, they shift to learning more from interaction with their peers (horizontal transmission). How such social learning processes promote individual adaptation has been the central theme in studies of cultural evolution (Cavalli-Sforza & Feldman, 1981; Boyd & Richerson, 1985, 1996; Kendal et al., 2018; Laland, 2004, 2017).

Previous studies on social learning have repeatedly shown that social learners performed better than asocial learners in information search (Kameda & Nakanishi, 2002, 2003; Mesoudi, 2011; Rendell et al., 2010; Toyokawa et al., 2014). Given limited time, individuals must strike an appropriate balance between exploring information on their own (i.e. asocial learning) and exploiting existing information by copying others’ choice behaviours (i.e. social learning) to maximise overall outcomes under uncertainty. This balance between asocial and social learning (which sometimes yields a game-theoretic, ‘dual exploration–exploitation dilemma’ at both the individual and group level; Toyokawa et al., 2014; Rogers, 1988) has mainly been studied in situations where agents in a population can observe and imitate each other's behaviour bilaterally and simultaneously.

However, this dilemma can also be a critical dimension in intergenerational cultural transmission, in which information is transmitted mostly unilaterally from predecessors to newcomers. If predecessors engage in suboptimal behaviours and leave more rewarding options unexplored, information search in the environment will remain incomplete and newcomers will still face the exploration–exploitation trade-off. Thus, newcomers need to balance between learning directly from preceding models and engaging in trial-and-error learning on their own. In other words, individuals must determine when to switch from exploiting information from their cultural parents to exploring new information by themselves. In line with this view, theoretical studies suggest that executing high-fidelity social learning followed by exploratory individual learning is one of the social learning strategies that can yield cumulative information across generations (Aoki, 2010; Borenstein et al., 2008). Developmental studies have also shown that children tend to be more exploratory than adults (Blanco & Sloutsky, 2024; Schulz, 2012), which may reflect the evolution of human life history, in which a distinctively long childhood allows a broad and intensive information search of the environment (Gopnik, 2020). Yet, to our knowledge, laboratory studies of individual strategies related to exploration–exploitation dilemmas in the context of intergenerational transmission have been rare.

**Figure 1.**
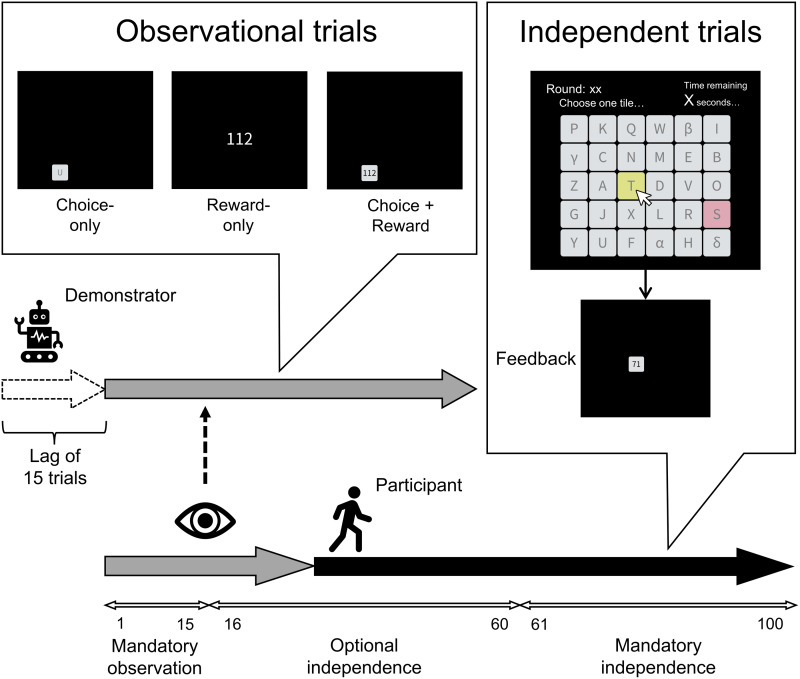
Overview of the experiment. Thirty options were arrayed in a 5 × 6 grid and displayed as 30 tiles with unique labels (e.g., P, K, Q) on a computer screen. Participants worked on the 30-armed bandit task for a total of 100 trials. The gray arrow illustrates trials in which participants observed the demonstrator's choice (the Choice-only condition), reward amount (the Reward-only condition), or both (the Choice-plus-reward condition) in each trial, without making their own choices. For those observational trials, the option selected by the demonstrator (tile U in this example) was revealed in the Choice-only and Choice-plus-reward conditions, while in the Reward-only condition, just the reward amount the demonstrator obtained was shown at the center. Observation was mandatory for the first 15 trials, but after this “mandatory observation” phase, participants could switch to independence at any trial (the “optional independence” phase: 16th-60th trial). After deciding to become independent or entering the “mandatory independence” phase (61st-100th trial), participants made their own choices without observation (the black arrow). The option that the participant chose in the previous trial was highlighted in pink.

Here, we address this issue in a simplified unilateral-transmission experiment in which a newcomer can utilise social information from a preceding demonstrator but not vice versa. We employ a multi-armed bandit (MAB) task (Fig. 1), which has been used extensively in reinforcement learning (Sutton & Barto, 2018), research on social learning (Deffner et al., 2020; McElreath et al., 2005, 2008; Toyokawa et al., 2019) and collective intelligence (Kameda et al., 2022). Although simpler and more abstract than the ‘cultural-product task’ (e.g. arrowheads: Mesoudi, 2011; wheel: Derex et al., 2019; stone flakes: Morgan et al., 2015), the MAB task enables us to make an unambiguous distinction between exploration and exploitation in information search under uncertainty – exploitation here means harvesting according to the most rewarding option based on one's own experience so far, while exploration means trying out other options that may possibly lead to better outcomes (Cohen et al., 2007; Hills et al., 2015; Sutton & Barto, 2018).

Besides the above standard definition, we argue that exploration–exploitation in the context of intergenerational transmission may involve additional aspects. In many real-world examples, people of younger generations and newcomers, who possess limited knowledge and skills, frequently undergo a phase of instruction and guidance from their elders and/or established group members (i.e. cultural parents) first, then transition to independent learning. Considering these sequences, two distinct aspects may characterise exploitation in intergenerational transmission: newcomers (a) continue to be fully ‘parasitic’ on cultural models and delay independence (i.e. they do not start to ‘live on their own’) or (b) simply adhere to the best-known option learned from observing the models’ choices, even after independence. Here, we examine how these two behavioural aspects may be related to the richness of social information provided by the cultural model. In the real world, social information can vary in richness (McElreath et al., 2008; Roberts & Goldstone, 2006; Toyokawa et al., 2014). Frequency of behavioural choices is arguably the most easily accessible information, as exemplified by e-commerce sites where information about customer choices (e.g. purchase frequencies, sales ranking) is often publicly available and can potentially be used to choose objectively better products. Information about the quality of a product can also sometimes be obtained through product reviews, consumer reports, and so on.

Thus, in this study, we examine how different types of social information available from a preceding demonstrator (i.e. experimental ‘cultural model’) may affect the behavioural strategies of participants (i.e. ‘newcomers’) concerning when to switch to independence (point (a) above) and their information search behaviours and performances after independence (point (b)). To address these points, we conducted an online behavioural experiment using a 30-armed bandit task. In the experiment, participants were paired with a computer-agent demonstrator that had made decisions preceding the participant and had acquired more knowledge about the environment (via the reinforcement learning algorithm to be explained in Methods). For their first 15 (out of 100) trials, participants simply observed the behaviours of the demonstrator and received rewards from the demonstrator's choices (Fig. 1). Thereafter, at any trial up to the 60th trial, participants could switch from observing and passively receiving rewards to making active independent choices. Once they had switched to independence, participants could not return to observing the demonstrator's choices. Note that, although the preceding demonstrator has more information about the environment than participants as newcomers, the demonstrator may still be in the process of learning and its knowledge may be incomplete. Therefore, uncertainty remains for participants about whether the demonstrator is exploiting the globally best option or is leaving better options unexplored.

Participants were randomly assigned to one of three conditions: the Choice-only condition, the Reward-only condition or the Choice-plus-reward condition. In the Choice-only condition, participants could only observe the option (‘arm’) chosen by the demonstrator from 30 alternatives but not the exact reward amount from the choice. In the Reward-only condition, participants could only observe the reward amount the demonstrator obtained from its choice, but not the option chosen by the demonstrator. In the Choice-plus-reward condition, participants were provided with both choice and reward information. Participants were explicitly instructed that the accumulated reward obtained by the demonstrator before their independence would later be included in their own compensation for the experiment. Thus, participants needed to determine when to shift from social learning (i.e. just observing the demonstrator's behaviour and receiving the same amount of rewards that the demonstrator obtained) to independence (i.e. making choices on their own without relying on the demonstrator's behaviour) to maximise their overall reward from 100 trials. We hypothesised that participants in the Reward-only condition would be slower in becoming independent from the demonstrator, compared with the participants in the Choice-only and the Choice-plus-reward conditions. This is because the participants in the Reward-only condition were able to infer the overall distribution of reward amount during observation but not the options which those rewards came from. Even though the participants were ‘fed by’ the preceding demonstrator, they could not learn how the demonstrator earned the rewards. In contrast, participants who had access to the choice information could guess the superior (i.e. more rewarding) options, as the options that the demonstrator repeatedly chose were likely to be better options (Kameda & Nakanishi, 2002, 2003; Naito et al., 2022; Rendell et al., 2010; Toyokawa et al., 2014). This information gap in the demonstrator information would yield a difference in the timing of the participants’ independence between the Reward-only condition and the other two conditions. Similarly, we also hypothesised that participants’ average behavioural performance after independence would be the highest in the Choice-plus-reward condition, followed by the Choice-only condition, and the lowest in the Reward-only condition, according to the difference in richness of the demonstrator information.

## 2. Methods

### 2.1. Participants

One-hundred and eighty-five students (67 females; mean age ± SD = 23.0 ± 3.9) from the subject pool at the University of Tokyo (Tokyo, Japan) and at Meiji Gakuin University (Tokyo, Japan) participated in an online experiment. The experiment was approved by and carried out in accordance with the guidelines and regulations of the ethics committee of the Department of Social Psychology at the University of Tokyo (UTSP-21021). All participants gave informed consent before the experiment. Upon finishing all of the trials, participants received compensation based on their performance during the experiment (mean ± SD = 1281 ± 126 JPY; 1 USD = 145 JPY).

### 2.2. Task design

Participants worked on a MAB task with 30 options for 100 trials. The 30 options were arrayed in a 5 × 6 grid on a computer screen (Fig. 1). The locations of the 30 options were fixed throughout the experiment and given unique labels (e.g. P, K, Q). For each chosen option in a trial, payoffs were randomly generated from a normal distribution with a mean of *μ* points and standard deviation of 20, rounded to the nearest non-negative integer (the equivalence of points to Japanese yen was not specified until the end of the experiment). Following Toyokawa et al. (2014), we set six categories for the 30 options in terms of the average quality (*μ*): 11 options were in the lowest-quality category with *μ* = 75, eight options with *μ* = 90, five options with *μ* = 105, three options with *μ* = 120, two options with *μ* = 135 and only one option was in the highest-quality category with *μ* = 150. This setting is similar to natural environments in which option quality is often negatively correlated with frequency (Kéfi et al., 2011). Participants were incentivised to maximise their cumulative rewards across 100 trials.

Each participant was paired with a computer agent (demonstrator) that made choices in the same 30 armed bandit task. As a ‘cultural parent’, the demonstrator had been learning the same environment ahead of the participant. Specifically, the demonstrator started its learning 15 trials earlier than the participant (see Fig. 1; ‘lag of 15 trials’). To set human-like parameters for the demonstrator, we conducted a pilot experiment in advance in which another set of participants (*N* = 91) worked on the same MAB task for 100 trials individually without social information. We then estimated participants’ choice parameters by fitting reinforcement learning models (Wilson & Collins, 2019; Sutton & Barto, 2018) and set the medians of the parameter estimates for the demonstrator's choice model in the main experiment. Thus, all participants were paired with the same demonstrator operating on the reinforcement-learning model with the same parameter values, although its actual choice behaviours were necessarily stochastic, and thus its choice history and reward amounts varied between participants. Alternatively, it was conceivable to randomly pair each participant in the main experiment with a participant in the pilot study as a demonstrator. However, as the participants’ choices in the pilot study varied owing to idiosyncratic behavioural characteristics plus the randomness inherent in the MAB task, this procedure would have introduced too much variability in terms of stimulus control into the main experiment. Therefore, we decided to create an ‘average person’ as a common (yet stochastic) demonstrator by fitting reinforcement learning models to data in the pilot study (for details about how we determined the choice model of the demonstrator and the length of precedence, see Supplementary Information, Section 1).

The experimental task was composed of three phases (Fig. 1). First, in the ‘mandatory observation’ phase, participants could only observe the choices of the demonstrator for 15 trials. As the demonstrator had started the task 15 trials ahead of the participant, the demonstrator's behaviour from its 16th to 30th trial was shown to the participant during the mandatory observation phase (see Supplementary Information for details). During the following ‘optional independence’ phase (16th–60th trial for the participant), participants could switch to making choices on their own at any trial; once participants had decided to be independent, they could no longer observe the demonstrator's behaviour. Lastly, the ‘mandatory independence’ phase started from the 61st trial. During this phase, all participants had to make choices on their own.

The participants were paid the sum of the rewards for all 100 trials, including the rewards that the demonstrator earned during the observation trials (until the 60th trial at the latest).

### 2.3. Conditions

There were three between-participants conditions that differed according to the demonstrator's information that was made available to the participants (Fig. 1). Participants were randomly assigned to the Choice-only condition (*n* = 62), the Reward-only condition (*n* = 62) or the Choice-plus-reward condition (*n* = 61). While observing the demonstrator's behaviour, participants in the Choice-only condition could only observe the option that the demonstrator chose in the trial. On the other hand, participants in the Reward-only condition could only observe the amount of reward that the demonstrator earned in the trial. In the Choice-plus-reward condition, participants could observe both the chosen option and the reward amount. This design allowed us to examine how different social information affects decisions of participants as a newcomer about when to become independent and their exploration strategies and performances after independence.

### 2.4. Experimental procedure

Participants accessed the experimental website individually with their own devices (only desktop or laptop computers were allowed). Before the experimental session began, participants were instructed that they would be paired with a demonstrator that had experienced the same environment ahead of them. We did not specify to the participants that the demonstrator was a computer agent operating on a reinforcement-learning model using the median parameter estimates of human participants in the pilot experiment.

After participants passed a comprehension test about the task, the main session started. After the main session, they answered a short questionnaire about their risk preferences (Holt & Laury, 2002; Eckel & Grossman, 2002), age, gender and other demographic information.

### 2.5. Statistical analyses

All the statistical analyses, including model fitting and simulations, were performed using R 4.2.3 (https://www.r-project.org) software. To compare the timing of independence across conditions, we conducted a log-rank test with the *survival* package. The log-rank test is commonly used in survival analysis to test the null hypothesis that there is no difference between the populations in the probability of an event (here, the decision of independence) at any time point (Bland & Altman, 2004). Other statistical tests were conducted with the *rstatix* package. Multiple comparison correction was conducted by adjusting *p*-values according to the Holm–Bonferroni method. Significance was set at *p* < 0.05. The Bayesian parameter estimation in the generalised linear mixed model was performed using the *brms* package. The models contained at least four parallel chains, and we confirmed that the Gelman–Rubin statistics were less than 1.1 and the effective sample sizes were greater than 1000 for all parameters.

## 3. Results

### 3.1. Timing of switch to independence

**Figure 2.**
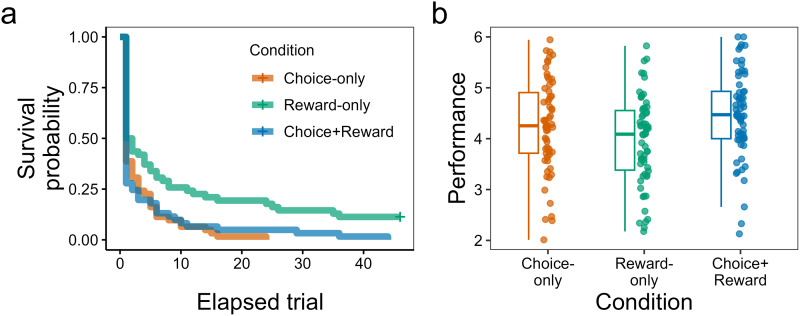
Timing of independence and behavioral performance thereafter. (a) Survival curves for the decision to continue observing the demonstrator's behaviors. The horizontal axis corresponds to the elapsed trial after the 15th trial (i.e., the last trial of the mandatory observation phase). The curve for the Reward-only condition does not reach zero, because there were participants who continued observation to the limit (the 60th trial), whereas all participants in the other two conditions switched to independence before the limit. (b) Behavioral performance after independence. We used the mean of the quality of options chosen by participants as a performance index, which ranges from 1 (choosing only the worst-category options) to 6 (choosing only the best-category option).

Figure 2a shows the timing of participants’ switches to independence as a survival curve. We found that 50% or more of the participants decided to become independent immediately after the mandatory observation phase was over (i.e. from the 16th trial), regardless of the type of social information (Fig. S2). At the same time, as we hypothesised, participants in the Reward-only condition tended to continue to rely on the demonstrator longer, with seven out of the 62 participants (11%) reaching the limit (i.e. the 60th trial). A log-rank test revealed that there was a significant difference in survival curves between the three conditions (*χ*^2^(2) = 14.48, *p* < 0.001). Pairwise comparisons of the survival curves yielded a significant difference between the Reward-only and Choice-only conditions (*χ*^2^(1) = 10.63, adjusted *p* = 0.003) and between the Reward-only and Choice-plus-reward conditions (*χ*^2^(1) = 9.16, adjusted *p* = 0.005), but not between the Choice-only and Choice-plus-reward condition (*χ*^2^(1) = 0.13, adjusted *p* = 0.722). These results indicate that participants tended to prefer to become independent as soon as possible but delayed the switch if the demonstrator's choices were not available.

### 3.2. Behavioural performance

Figure 2b shows the average behavioural performance of participants after independence. We scored behavioural performance in each trial based on the quality of chosen options from 1 (the 11 lowest-quality options) to 6 (the single highest-quality option). As we hypothesised, the average behavioural performance was in accordance with the richness of the demonstrator information, i.e. the highest in the Choice-plus-reward condition, followed by the Choice-only condition, and the lowest in the Reward-only condition. One-way analysis of variance (ANOVA) indicated that there was a significant difference between the three conditions (*F*(2, 182) = 5.40, *p* = 0.005, *η_p_*^2^ = 0.06). A *post-hoc* multiple comparison test revealed that participants in the Choice-plus-reward condition (*M* = 4.48) performed better than in the Reward-only condition (*M* = 3.96; adjusted *p* = 0.004), whereas there was no difference between the Choice-plus-reward and the Choice-only conditions (*M* = 4.26; adjusted *p* = 0.171). These results imply that informational benefits for the participant originate mainly from the demonstrator's choice history rather than the reward amount.

**Figure 3.**
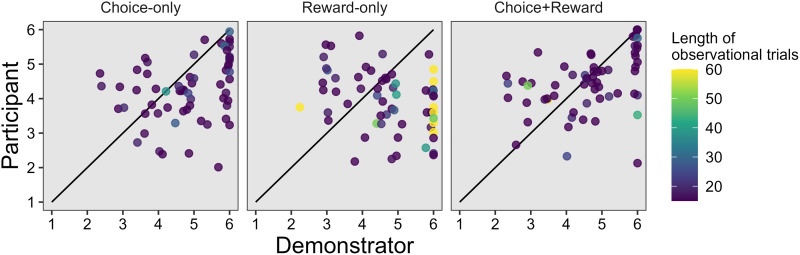
Pair-level comparison of behavioral performance between demonstrators and participants. X-axis refers to the paired demonstrator's performance (averaged across trials) after the participant switched to independence. Y-axis refers to the participant's performance during the same independent trials. Dot colors indicate the length of observational trials of each participant, with the lighter color meaning longer observations. Diagonal lines correspond to the cases where performance matches within the pair.

Then, how did the participants, performance compare with the demonstrator's performance? Figure 3 shows the average performance of each participant after independence, along with the average performance of the paired demonstrator during the same period (from the participant's independence to the end of the task). Since the demonstrator continued to make its own choices even after the participant became independent, the performance for the same period (i.e. trials after the participant's independence) can be compared between the demonstrator and the participant. If participants had extracted the same level of information about the search space through observation as the paired demonstrator had learned itself, they should have performed at least no worse than the demonstrator after independence. However, as seen in Figure 3, participants’ performance was lower than the paired demonstrator's in the Choice-only condition (*t*(61) = −3.80, *p* < 0.001, *d* = −0.57, 95% CI [−0.89, −0.27]) and in the Reward-only condition (*t*(61) = −3.57, *p* < 0.001, *d* = −0.74, 95% CI [−1.21, −0.28]). Participants in the Choice-plus-reward condition also slightly underperformed the demonstrator, although the difference was not significant (*t*(60) = −1.08, *p* = 0.283, *d* = −0.15, 95% CI [−0.42, 0.13]). Such divergence of performance between participant and demonstrator was also confirmed visually in the learning curves for each pair (see Figs S3–S5), which indicates that participants largely failed to perform as well as the demonstrator after independence. Notably, even in the Choice-plus-reward condition, where the participants received exactly the same information as the demonstrator, they did not outperform the demonstrator after independence. Taken together, we found no evidence for cumulative improvement in performance across the two ‘generations’.

### 3.3. Use of social information and participants’ own exploration after independence

Recall that 50% or more of the participants became independent immediately after the mandatory observation phase was over and started learning on their own (Fig. S2). Observing the demonstrator's choices for a longer period could have enabled participants to gather more information about the environment in order to earn a higher reward on average; such early independence may have resulted in insufficient accumulation of social information (Morin et al., 2021), leading to inferior performance compared with the demonstrator (Fig. 3). To check this possibility, we conducted several analyses of participants’ choice behaviours after independence.

**Figure 4.**
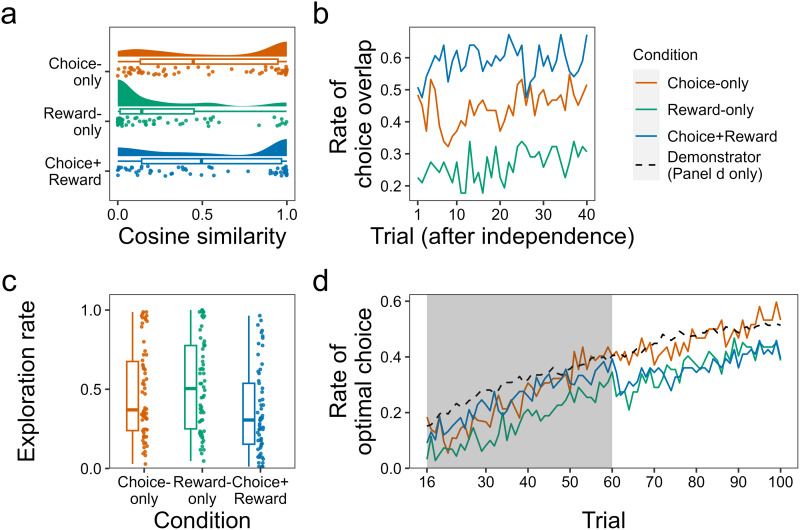
Participants generally exhibited exploratory behavior after independence. (a) Cosine similarity of the choice proportions among the 30 options between each pair. This value represents overall “option-by-option” choice similarity between the demonstrator and the participant (i.e., participant's after-independence imitation of the demonstrator's choices during the observation trials), ranging from 0 (not similar at all) to 1 (highly similar). (b) Behavioral similarity between a participant and the demonstrator during the first 40 trials after independence. We checked whether a participant chose any of the options that the demonstrator had chosen during the observation period in each trial. The graph shows the proportions of the participants who exhibited such behavioral similarity in each trial. (c) Exploration rates. Each dot refers to the proportion of exploratory choices in which a participant selected options other than the best-known option (i.e., the option with which she/he had directly experienced the largest mean reward up to the preceding trial) after independence. (d) Change in the proportion of participants who chose the optimal (single-best) option. The proportion of demonstrators choosing the optimal option (i.e., the demonstrator's continued improvement during the mandatory independence phase, with the time lag adjusted) is also shown for comparison (black line).

First, to measure participants’ overall fidelity to the demonstrator, we examined the similarity between the demonstrator's choices during observation and the participant's own choices after independence. For each pair, we calculated the cosine similarity between two vectors of length 30, whose elements correspond to the proportions of choosing each of the 30 options (see Figs S6–S8). This index shows overall ‘option-by-option’ fidelity of the participant's choices after independence to the demonstrator's behaviours during observation. Figure 4a shows the distribution of the cosine similarity by condition. Not surprisingly, the distribution of similarity in the Reward-only condition, where the choice history of the demonstrator was absent, was right skewed compared with that in the Choice-only (Kolmogorov–Smirnov test: *D* = 0.35, adjusted *p* = 0.002) and the Choice-plus-reward (*D* = 0.32, adjusted *p* = 0.003) conditions. However, even in the Choice-plus-reward condition where participants received exactly the same information as the demonstrator during observation, a sizable proportion of participants exhibited low similarities (e.g. 51% of the participants exhibited less than 0.5 similarity), indicating that the fidelity in copying across generations was not high. Low fidelity was also evident in the Choice-only condition.

Next, to examine temporal changes in similarity between the demonstrator and a participant, we checked whether a participant chose any of the options that the demonstrator had chosen during observation. Here, we focused on the first 40 trials after a participant switched to independence because all participants (including those who continued observation until the mandatory independence phase: Fig. 1) performed at least 40 trials independently. Figure 4b displays ‘trial-by-trial’ proportions of the participants who exhibited behavioural similarity to the demonstrator. In line with the results from Figure 4a, the similarity between the participants and the demonstrator was generally modest. We fitted a logistic mixed model with random intercept for each participant to predict whether a participant chose any of the options that the demonstrator had chosen during observation (*y* = 1) or not (*y* = 0) with the conditions and trials. The fixed effect of condition (the Choice-only vs. Choice-plus-reward condition) was negative and statistically significant (*β* = −1.04, 95% CI [−1.82, −0.25]), which indicates that participants in the Choice-only condition were generally more exploratory right after independence, trying out new options that had not been chosen by the demonstrator. Additionally, the fixed effect of trials was positive (*β* = 0.14, 95% CI [0.08, 0.21]), which means that the behavioural similarity with the demonstrator generally increased over trials. This is likely to have resulted from participants’ own explorations through which they gradually settled on the same options that the demonstrator had chosen.

We next examined participants’ individual exploration tendencies after independence, irrespective of the demonstrator's choices. Here, based on the standard definition of exploration (Sutton & Barto, 2018), we checked the proportion of choices in which a participant selected options other than the best option among their already-chosen options (the option with which she/he had directly experienced the largest mean reward up to the preceding trial; that is, the best-known option not by observation of the demonstrator but experienced directly via their own choice). As seen in Figure 4c, there was a significant overall difference in exploration rates across the conditions (Kruskal–Wallis test: *χ*^2^(2) = 11.48, *p* = 0.003, *ɛ*^2^ = 0.05). Again, not surprisingly, the exploration rate was highest in the Reward-only condition. Notably, however, participants in the Choice-only condition also engaged in more exploratory choices than those in the Choice-plus-reward condition (Wilcoxon rank-sum test: *W* = 2375, adjusted *p* = 0.029). Although the timing of independence was indistinguishable between the two conditions (Fig. 1a), participants who could observe the demonstrator's choices but not the reward amounts explored more after independence. In addition, we analysed participants’ exploration rates based on a combined benchmark with the best option demonstrated during observation and the best option directly experienced after independence. Participants’ exploration rates based on the combined benchmark were generally higher than those based only on the direct-experience benchmark. However, the overall pattern with the combined benchmark was not different from that of the original benchmark; the average exploration rate was highest in the Reward-only condition, moderate in Choice-only, and lowest in Choice-plus-reward (see Fig. S9 for details).

Lastly, how did such differences in the exploration tendency affect participants’ choice results, especially the likelihood of finding the objectively optimal option? Figure 4d shows the proportion of participants choosing the single-best (*μ* = 150) option after independence in comparison with the demonstrator's choices during the same period. We fitted a logistic mixed model with random intercepts for each participant, which predicts whether a participant chose the best option (*y* = 1) or not (*y* = 0) during the mandatory independence phase (i.e. the last 40 trials). As seen in the figure, the participants in the Choice-only condition found the optimal option equally well to the demonstrator (*β* = −0.12, 95% CI [−2.88, 2.64]). In contrast, the participants in the Choice-plus-reward condition performed worse than the demonstrator (*β* = −3.06, 95% CI [-5.94, −0.27]). Recall that, compared with the participants in the Choice-plus-reward condition, those in the Choice-only condition were less loyal to the demonstrator's choices (Fig. 4b) and explored more (Fig. 4c) after independence. The results in Figure 4d indicate that they eventually caught up with the demonstrator in identifying the best option through their own explorations, while the participants in the Choice-plus-reward condition were left behind by the demonstrator's continued improvement during the mandatory independence phase.

## 4. Discussion

Social learning within and across generations is one of the core human capacities that has helped us to develop highly sophisticated cultures and expand our populations throughout the world (Boyd & Richerson, 1985, 1996; Cavalli-Sforza & Feldman, 1981; Kendal et al., 2018; Laland, 2017; Tennie et al., 2009; Tomasello, 1999). Although intergenerational information transmission is ubiquitous, little is known about when and how people switch from learning loyally from their preceding models (e.g. cultural parents) to choosing independently through their own explorations. To address this question, we conducted a simplified unilateral-transmission experiment whereby a newcomer can utilise social information from a preceding demonstrator (but not vice versa), and employed a MAB task, which has been used as a common platform to investigate social learning strategies (e.g. McElreath et al., 2008; Roberts & Goldstone, 2006; Toyokawa et al., 2014; Kameda et al., 2022).

This study examined two dimensions of the participants’ dependence on their paired demonstrator. Firstly, we focused on the extent to which the participants continued to be ‘parasitic’ on the demonstrator (i.e. passively letting the demonstrator's choices determine their own payoffs: Fig. 1). The results showed that more than 50% of the participants were minimally parasitic, switching to independence right after the mandatory observation phase (Fig. 2a). Such an early-independence tendency among the participants may be seen as an ‘action bias’ (people prefer action rather than inaction even though there is no clear indication that it brings a better result: Patt & Zeckhauser, 2000), and also probably reflected the boredom of merely observing the demonstrator's behaviours in the experiment. Yet, as we had hypothesised, the participants in the Reward-only condition chose to be dependent on the demonstrator significantly longer than the other two conditions (Choice-only and Choice-plus-reward), with seven out of 62 (11%) reaching the limit (Fig. S2). This suggests that the participants strategically capitalised on the information from the demonstrator and that the choice information was more useful than the reward information for switching to independence (Fig. 2a) and better behavioural performance after independence (Fig. 2b).

Secondly, we investigated the degree of dependence at the level of informational influence that the participants received from the demonstrator during observation. We assessed participants’ information search strategies after independence by quantifying how their behavioural performances and choice patterns were similar to or diverged from those of the demonstrator. As shown in Figure 3, comparisons of the participants’ behavioural performance after independence with the paired demonstrator's performance during the same period showed an intriguing pattern. Generally, participants underperformed the demonstrator after independence, which seems to imply that the participants did not effectively utilise the social information from the demonstrator after independence. In line with this speculation, post-independence participants tended to explore options that their demonstrator had not chosen (Figs. 4a and 4b). Also, as reported in the Supplementary Information (Figs. S3–S5), the learning (performance) curves of participants after independence did not necessarily ‘extend’ the pre-independence learning curve of the paired demonstrator continuously.

These results are in line with previous research on cultural transmission that showed people often underutilise social information from peers and excessively rely on individual learning. While it remains unclear what proximate or ultimate factors lay behind such ‘egocentric discounting’ (Morin et al., 2021), it is important to note that we have demonstrated this phenomenon experimentally in an intergenerational transmission context where participants could freely choose how long they continued to rely on the preceding demonstrator. The demonstrator, who was ahead of the participants by 15 trials (Fig. 1), displayed fairly refined behaviours to participants; the demonstrator had learned about the environment better than the participants (through its own trial-and-error during its first 15 trials; see Supplementary Information, Section 1). Yet, in the experiment, the participants seem to have underappreciated the superior knowledge of the demonstrator, perhaps deeming that its behaviour was biased towards exploitation (i.e. harvesting according to the most profitable option based on current knowledge) without sufficient exploration. These results seem to indirectly support the idea that explicit teaching and verbal communication to convey information about the model's expertise and behaviour, which is often difficult to obtain only through passive observation, plays a key role in cumulative cultural evolution of humans (Caldwell et al., 2018; Csibra & Gergely, 2009, 2011; Dean et al., 2012; Derex et al., 2013; Fogarty et al., 2011; Kameda et al., 2022; Lucas et al., 2020; Morgan et al., 2015; Osiurak et al., 2023). Furthermore, a recent neurocognitive experiment has also shown that bilateral interaction, which was absent in the current unilateral setting (i.e. information flowed only from the demonstrator to the participant), is critical for emergence of social norms and long-lasting socially shared realities (Kuroda et al., 2022; Naito et al., 2022). An important direction for future research is to clarify how such bilateral interaction (e.g. Csibra & Gergely, 2009, 2011) may facilitate adaptive use of social information under the exploration–exploitation trade-off in the context of intergenerational transmission.

We also found that, after independence, participants in the Choice-plus-reward condition engaged in exploration (i.e. selecting options other than the best-known option that she/he had directly experienced up to the preceding trial) less frequently than those in the Choice-only condition (Fig. 4c). Enriching transmitted information contributed to facilitating social information use (Fig. 4b), but this also entailed the risk of being stuck in a suboptimal solution; in contrast, those with choice-information only eventually caught up with the demonstrator's continued improvement, identifying the optimal option through their own explorations (Fig. 4d). Interestingly, Derex et al. (2019) reported that transmission of detailed causal information constrains learners’ exploration in the solution space for creating a physical artifact. It has also been reported that allowing participants to observe others’ evaluations of chosen options as well as choice-frequency information had a detrimental impact on performance in collective information foraging (Toyokawa et al., 2014). In sum, learners’ performance may not always monotonically improve as transmitted information becomes richer. Although this study could not explore the cognitive mechanisms underlying this phenomenon and their longitudinal population-level outcomes (i.e. cumulative culture), computational modelling (Wilson & Collins, 2019) that directly deals with the richness of transmitted information and agent-based simulations that extrapolate from the experimental results may be useful in future research.

Cognitive strategies involving exploration–exploitation trade-offs under social influence, which we investigated with a simplified and controlled setting, may have implications for cultural variations in the timing of economic independence of younger people. In traditional cultures, social norms often dictate that younger individuals join the workforce early as apprentices and learn from adults (Fongnzossie et al., 2023). Younger individuals in industrial societies, on the other hand, are often under the economic protection of parents for a longer period (Arnett, 2000; Nettle & Frankenhuis, 2020). For example, sociological studies have shown that, in Western European countries, higher socio-economic status of parents is related to their children's delay of entry into first marriage or co-residential union (Brons et al., 2017; Wiik, 2008). We believe that examining individual strategies involving exploration–exploitation trade-offs could help clarify intergenerational (arguably game-theoretic) dynamics (e.g. Toyokawa et al., 2014; Rogers, 1988) that underlie such societal variations.

There are several limitations in this study. Firstly, we focused on the exploration–exploitation trade-off in a simple and narrow search space using the MAB task. The advantage of using such artificial tasks is that the optimality of behaviour and the distinction between exploration and exploitation can be clearly defined, and cognitive processes underlying social learning can be analysed by computational models (McElreath et al., 2005, 2008; Toyokawa et al., 2017, 2019; Najar et al., 2020). However, one could note that such artificial tasks do not reflect actual rich environments with multidimensional features (Miton & Charbonneau, 2018). The MAB situation also does not address the potential exponential increase in available behavioural options through innovations and combinations of ideas (Derex, 2022; Mesoudi & Thornton, 2018). We believe that there is a trade-off between increasing task complexity and understanding computational bases of social learning as enabled in simplified tasks. Addressing this trade-off (FeldmanHall & Nassar, 2021; Wise et al., 2023) will be one of the key methodological issues in future experimental studies on cultural evolution.

Secondly, this study does not address individual differences in social information use. Although we tried to develop several computational models to shed light on possible individual differences in information use, the model analysis was not successful, mainly owing to the stochastic nature of the demonstrator's behaviour. Many variants of social information use, such as anti-conformity and contrariness, have been documented in the literature (Efferson et al., 2008; Mesoudi, 2011; Morgan et al., 2012; Toyokawa et al., 2019), suggesting possible sensitivity of human social learning to contextual variables (e.g. socio-economic status) and other developmental factors (e.g. Gergely et al., 2002; Mesoudi et al., 2015; Nettle, 2019). Further research is needed to clarify how heterogeneity in social learning strategies among people may affect cultural evolution.

In summary, our results showed that the participants adopted different strategies about when to stop observational learning and exhibited different behavioural patterns after independence, depending on what type of information (i.e. choice history and/or reward amount) was available from the preceding demonstrator (i.e. cultural model). At the same time, they typically failed to make the best use of social information after independence. This tendency could undermine cumulative improvement in performance under the exploration–exploitation trade-off across generations. We believe that these findings have implications not only for understanding the building blocks of social learning but also for designing effective intergenerational information-transmission systems in scientific research and education.

## Supporting information

Suganuma et al. supplementary material Suganuma et al. supplementary material

## Supplementary material

To view supplementary material for this article, please visit https://doi.org/10.1017/ehs.2024.29

## Research transparency and reproducibility

This study is preregistered in Open Science Framework. Prior to the present experiment, we ran another experiment using a similar MAB setup (https://osf.io/wpr5g). However, the demonstrator that we used in that experiment had a perfect memory (no forgetting), exhibiting unrealistic, overly efficient behaviours as a cultural model. Based on this reflection, we added the forgetting parameter to the model of the preceding demonstrator and ran the present experiment (https://osf.io/gx83z) by pairing each participant with the modified demonstrator. The data and code associated with this research are available online (https://osf.io/z8yd9).
