# Supplementary material for: When to stop social learning from a predecessor in an information-foraging task
Source: Evol Hum Sci. 2025 Jan 20;7:e2. doi: 10.1017/ehs.2024.29 (PMC11810515; doi:10.1017/ehs.2024.29)
Supplement: Suganuma et al. supplementary material [file S2513843X2400029Xsup001.pdf]

Supplementary Information for
When to stop social learning from a predecessor in an information-foraging task

Hidezo Suganuma[1*] Aoi Naito[2, 3] Kentaro Katahira[4] Tatsuya Kameda[1, 5, 6, 7]

[1] Department of Social Psychology, The University of Tokyo, 7-3-1 Hongo, Bunkyo-ku, Tokyo 113-0033, Japan

[2] School of Environment and Society, Tokyo Institute of Technology, 3-3-6 Shibaura, Minato-ku, Tokyo 108-0023, Japan

[3] Japan Society for the Promotion of Science, 5-3-1 Kojimachi, Chiyoda-ku, Tokyo 102-0083, Japan

[4] Human Informatics and Interaction Research Institute, National Institute of Advanced Industrial Science and Technology (AIST), Tsukuba, Ibaraki 305-8566, Japan

[5] Faculty of Mathematical Informatics, Meiji Gakuin University, 1518 Kamikuratachou, Totsuka-ku, Yokohama, 244-8539 Japan

[6] Center for Experimental Research in Social Sciences, Hokkaido University, N10W7, Kita-ku, Sapporo, Hokkaido 060-0810, Japan

[7] Brain Science Institute, Tamagawa University, 6-1-1 Tamagawagakuen, Machida, Tokyo, 194-8610 Japan

*Corresponding author: Hidezo Suganuma (suga-zo0528utokyo@g.ecc.u-tokyo.ac.jp)

Table of Contents

1. The demonstrator's (computer agent's) model *3*

1.1. Overview 3

1.2. Models 3

1.3. Fitting procedure 5

1.4. Modelling results 5

2. Timing of independence *8*

3. Individual learning curves of all demonstrator-participant pairs *9*

4. Proportions of choosing each of the 30 options *13*

5. Exploration rates under an alternative definition *17*

References *18*

1. The demonstrator's (computer agent's) model

1.1. Overview

To set human-like parameters for the demonstrator (computer agent), we conducted a pilot experiment in which another set of participants worked on the same task online. Ninety-one students at the University of Tokyo (32 females; Mean age ± S.D. = 23.0 ± 1.9) worked on the same 30-armed bandit task as used in the main experiment for 100 trials individually without social information. After finishing the experiment, participants were compensated according to their task performances (Mean ± S.D. = 1284 ± 119 JPY).

1.2. Models

To develop a model that represents the average behavior of human participants reasonably well, we fitted a series of reinforcement learning models (Sutton & Barto, 2018; Wilson & Collins, 2019) to the behavioral data and estimated behavioral parameters for each participant. In the full model, participants update the expected reward of option k in response to reward r according to the Rescorla-Wagner learning rule:

$$Q_{t+1}(k) = Q_t(k) + \alpha\big(r_t - Q_t(k)\big), \tag{1}$$

where α is the learning rate that takes a value between 0 to 1. We treated the initial value of each option, $Q_0 = q_{\text{init}}$, as a free parameter. In order to take account of forgetting effect, we assumed that the values of unchosen options $\bar{k}$ return to the initial value according to the following equation:

$$Q_{t+1}(\bar{k}) = Q_t(\bar{k}) + (1 - \alpha_{\text{f}})\left(q_{\text{init}} - Q_t(\bar{k})\right), \tag{2}$$

where α_{f} is the forgetting rate. This is a free parameter which ranges from 0 to 1. When $\alpha_{\text{f}} = 1$, there

is no forgetting effect. When $\alpha_{\mathrm{f}} = 0$, the values of unchosen options immediately return to the initial value.

The overall valuation function consists of the expected reward and the correction term of the UCB1 (Upper Confidence Bound) score as follows:

$$V_t(k) = Q_t(k) + \tau \cdot \sqrt{\frac{\log t}{T_t(k)}}, \tag{3}$$

where $T_t(k)$ is the frequency of choosing option k so far, and $\tau(> 0)$ is a free parameter that governs the degree to which uncertainty is prioritized relative to the expectations of reward. τ can be interpreted as the uncertainty premium of each participant. In other words, when τ is greater, exploration becomes more directed to novel options.

The probability of choosing option k is produced according to the softmax choice rule:

$$P_t(k) = \frac{\exp\left(\beta \cdot V_t(k)\right)}{\sum_{i=1}^{30} \exp\left(\beta \cdot V_t(i)\right)}, \tag{4}$$

where $\beta(> 0)$ is the 'inverse temperature' parameter that controls the level of stochasticity in the choice, ranging from $\beta = 0$ for completely random responses to $\beta = \infty$ for deterministically choosing the option with the highest value.

Overall, the full model has five free parameters: α, α_{f}, β, τ, and q_{init}. The prior distributions for each parameter were defined as follows:

$$\alpha \sim \mathrm{Beta}(2,2) \tag{5}$$

$$\alpha_{\mathrm{f}} \sim \mathrm{Beta}(2,2) \tag{6}$$

$$\beta \sim \mathrm{Gamma}(2,1/3) \tag{7}$$

$$\tau \sim \mathrm{Cauchy}^{+}(0,10) \tag{8}$$

$$q_{\text{init}} \sim \text{Gamma}(9.5,10) \tag{9}$$

We considered seven sub-models in addition to this full model (see Table S1). These models include those in which q_{init} is fixed at 95 (i.e., the expected reward for one random choice), those in which τ is fixed at 0 (so the participant's uncertainty premium is not considered), and those in which α_{f} is fixed to 1 (so there is no forgetting effect). By comparing these models, we aimed to identify a computational model that simulates the behaviours of human participants reasonably well.

1.3. Fitting procedure

We estimated individual parameters with the Markov Chain Monte Carlo (MCMC) method using Stan 2.31.0 (https://mc-stan.org) in R. The models contained at least four parallel chains, and we confirmed convergence of the MCMC with the Gelman-Rubin statistics $\hat{R} < 1.1$. We calculated WBIC from MCMC samples for each model-participant combination and determined the approximate goodness of fit for each model by summing these values for all participants. To identify the globally best-fitting model, we derived the Widely Applicable Bayesian Information Criterion (WBIC; Watanabe, 2013) for each participant-model pair from the MCMC samples.

1.4. Modelling results

The result of the model comparison is summarized in Table S1. According to the sum of estimated WBICs, the full model (No. 8) fit the behavioural data best. We then calculated the median of the maximum a posterior probability (MAP) estimates of the five behavioural parameters under the full model and designated the median estimates as the behavioural parameters of the demonstrator to be implemented in the main experiment. We confirmed that the performance of the computer agent (demonstrator) is approximately equivalent to that of the average human participant in the pilot experiment ($t(210) = 1.26$, $d = 0.16$, 95% CI [-0.09, 0.40], $p = .209$; Fig. S1).

Table S1. Result of the model comparison. The middle three columns indicate which free parameters were included in each model.

Model	q_{init}	τ	α_{f}	Sum of WBIC
1				14475
2	x			13587
3		x		14312
4	x	x		13503
5			x	14451
6	x		x	13443
7		x	x	14421
8 (full model)	x	x	x	13282

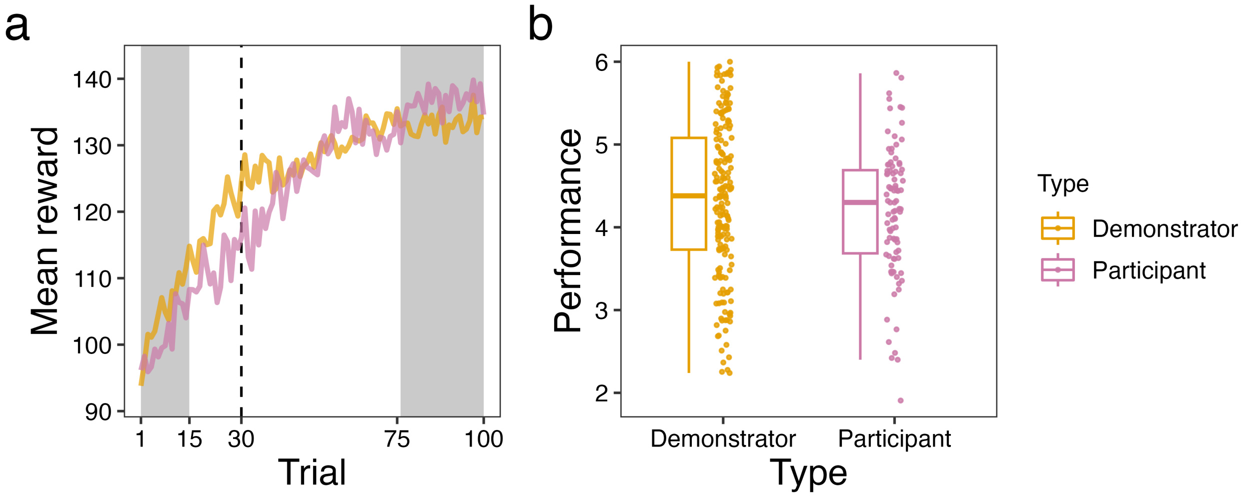

Fig. S1. Behavioral performance of the demonstrator. (a) Learning curves of the demonstrator (computer agent) and human participants. Each curve shows mean reward of the 185 demonstrators implemented in the main experiment, and mean reward obtained by the 91 participants in the pilot experiment. Trials in which the participants in the main experiment could not observe the preceding demonstrator's behavior were shaded in gray. The vertical dashed line indicates the trial (i.e., the 30th trial for the demonstrator) after which the participants in the main experiment could switch to independent trials any time. (b) Behavioral performance of the demonstrator (in the main experiment) and the participants in the pilot experiment, in terms of the quality of chosen option, which ranges from 1 (choosing only the worst-category options) to 6 (choosing only the best-category option). There was no significant performance difference between the demonstrators and the participants ($t(210) = 1.26$, $d = 0.16$, 95% CI [-0.09, 0.40], $p = .209$).

2. Timing of independence

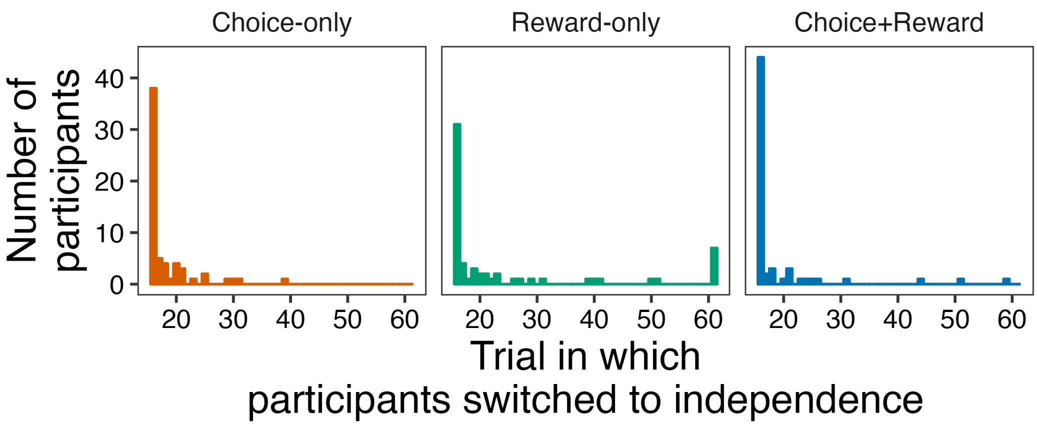

Fig. S2. Distribution of the timing of independence. 38 (61.3%) participants in the Choice-only condition, 31 (50.0%) in the Reward-only condition, and 44 (72.1%) in the Choice-plus-reward condition switched to independence right after the mandatory independence phase was over (i.e., the 16th trial).

3. Individual learning curves of all demonstrator-participant pairs

Individual learning curves of all participant-demonstrator pairs are shown in Figs. S3-5. The x-axis refers to 100 trials in the experiment (Fig. 1). The solid lines are derived from nonparametric regression analysis with locally estimated scatterplot smoothing (LOESS).

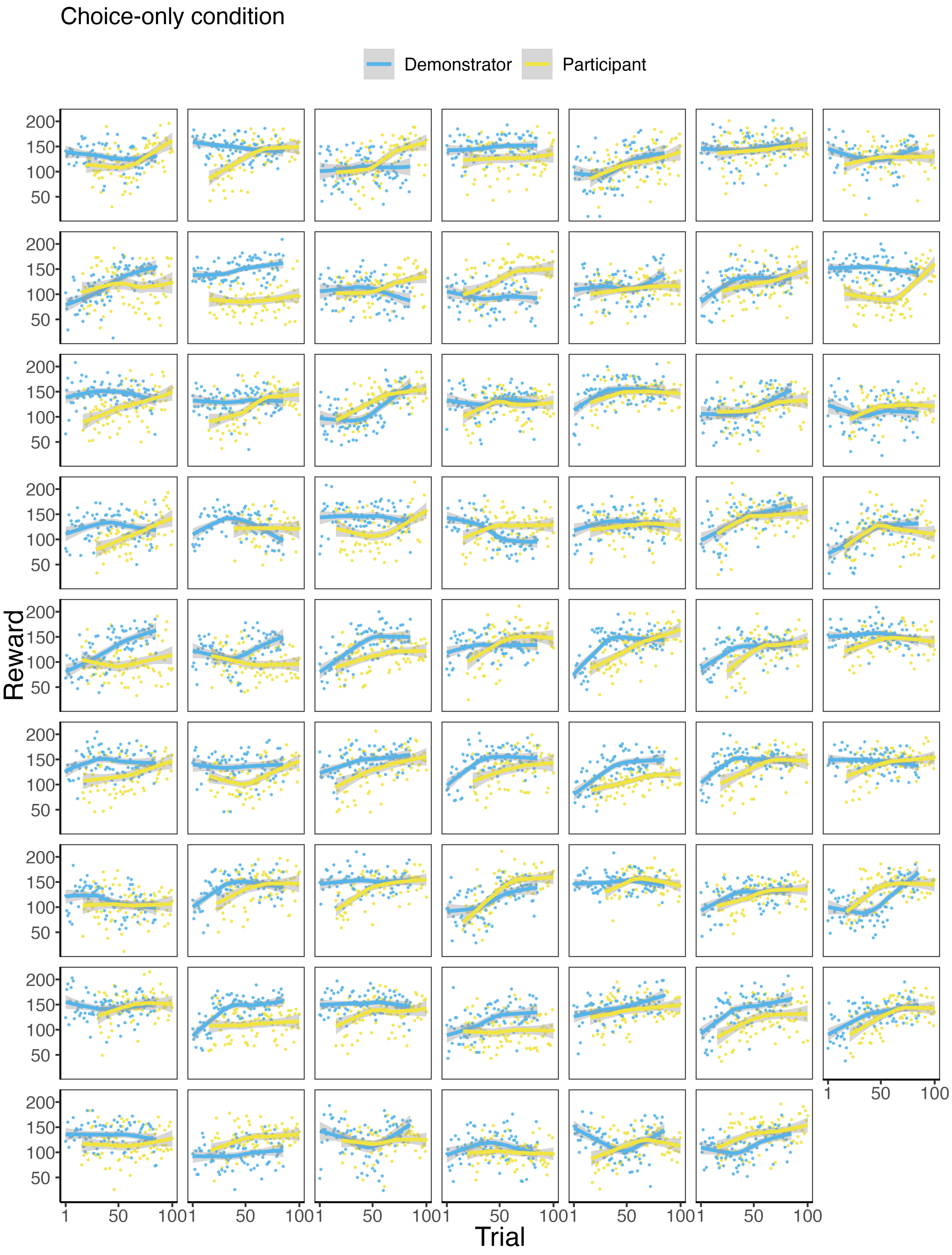

Fig. S3. Individual learning curves of all demonstrator-participant pairs (Choice-only condition).

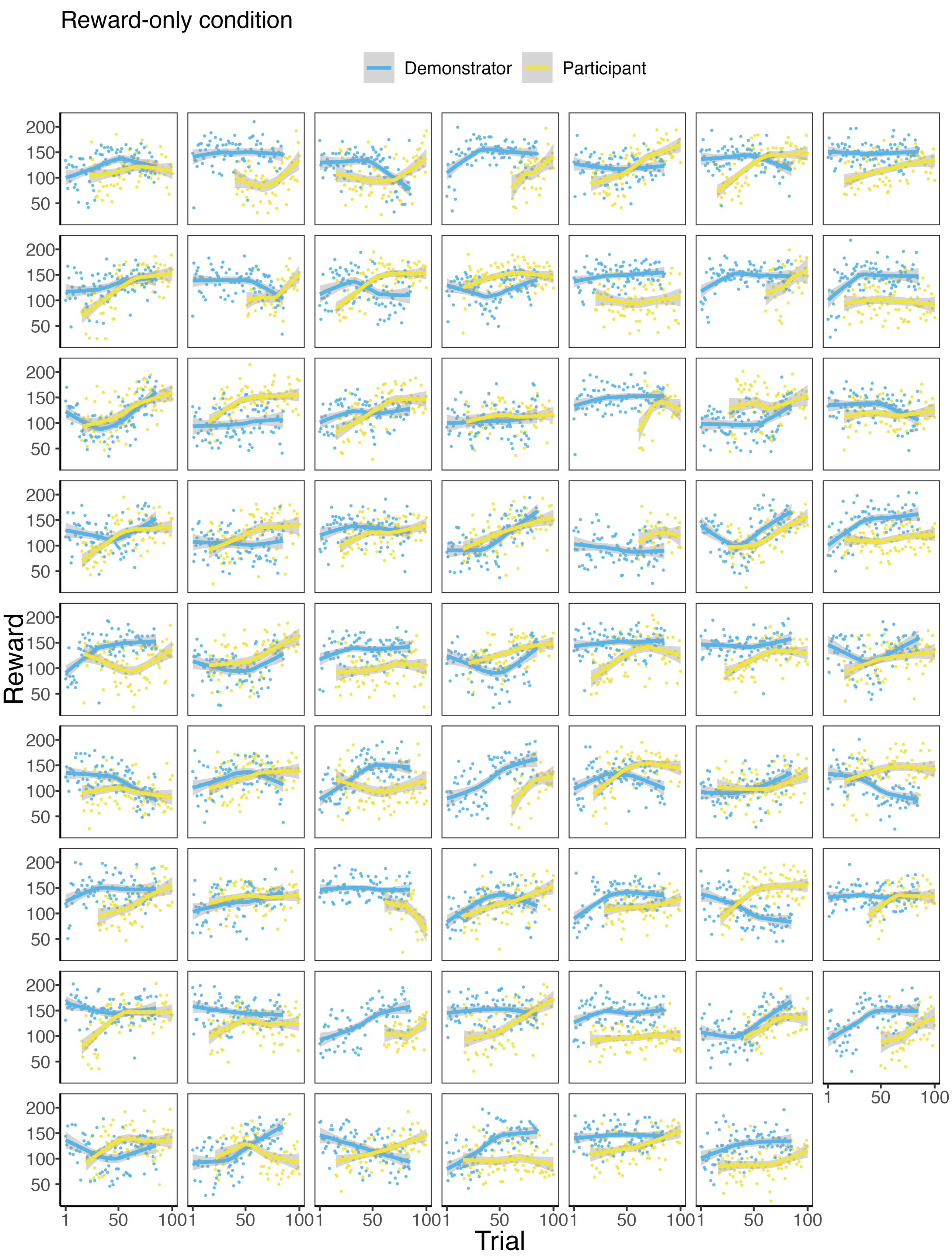

Fig. S4. Individual learning curves of all demonstrator-participant pairs (Reward-only condition).

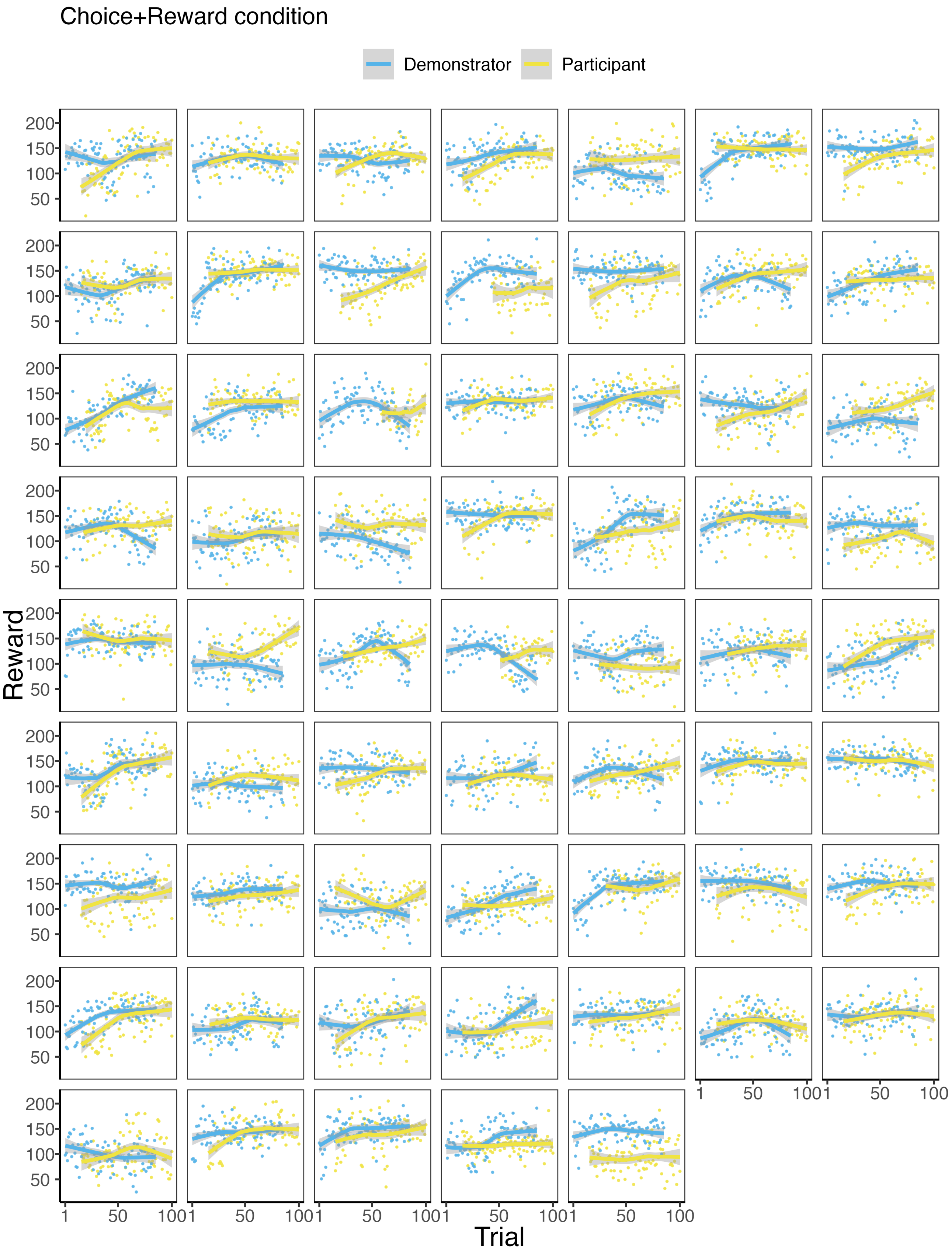

Fig. S5. Individual learning curves of all demonstrator-participant pairs (Choice-plus-reward condition).

4. Proportions of choosing each of the 30 options

Figs. S6-8 show proportions of the demonstrator's choices in observational trials (left) and the participant's choices in independent trials (right) over the 30 options. The number above each panel indicates the IDs of the demonstrator-participant pairs in each condition. The locations of the 30 options were identical to those in the experiment. The number in each grid represents the average reward of the option.

Choice-only condition

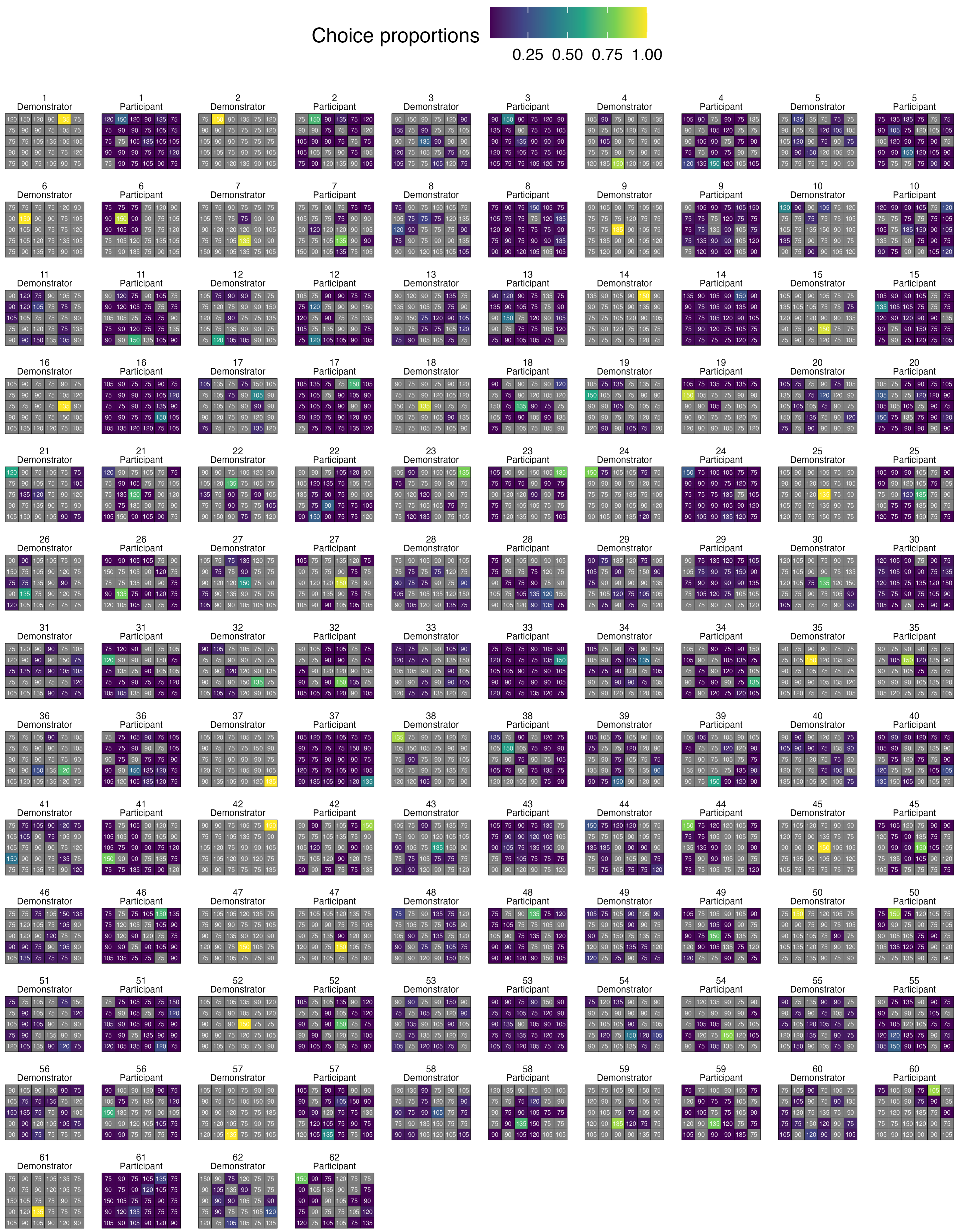

Fig. S6. Proportions of the demonstrator's choices in observational trials (left) and the participant's choices in independent trials (right) over the 30 options (Choice-only condition).

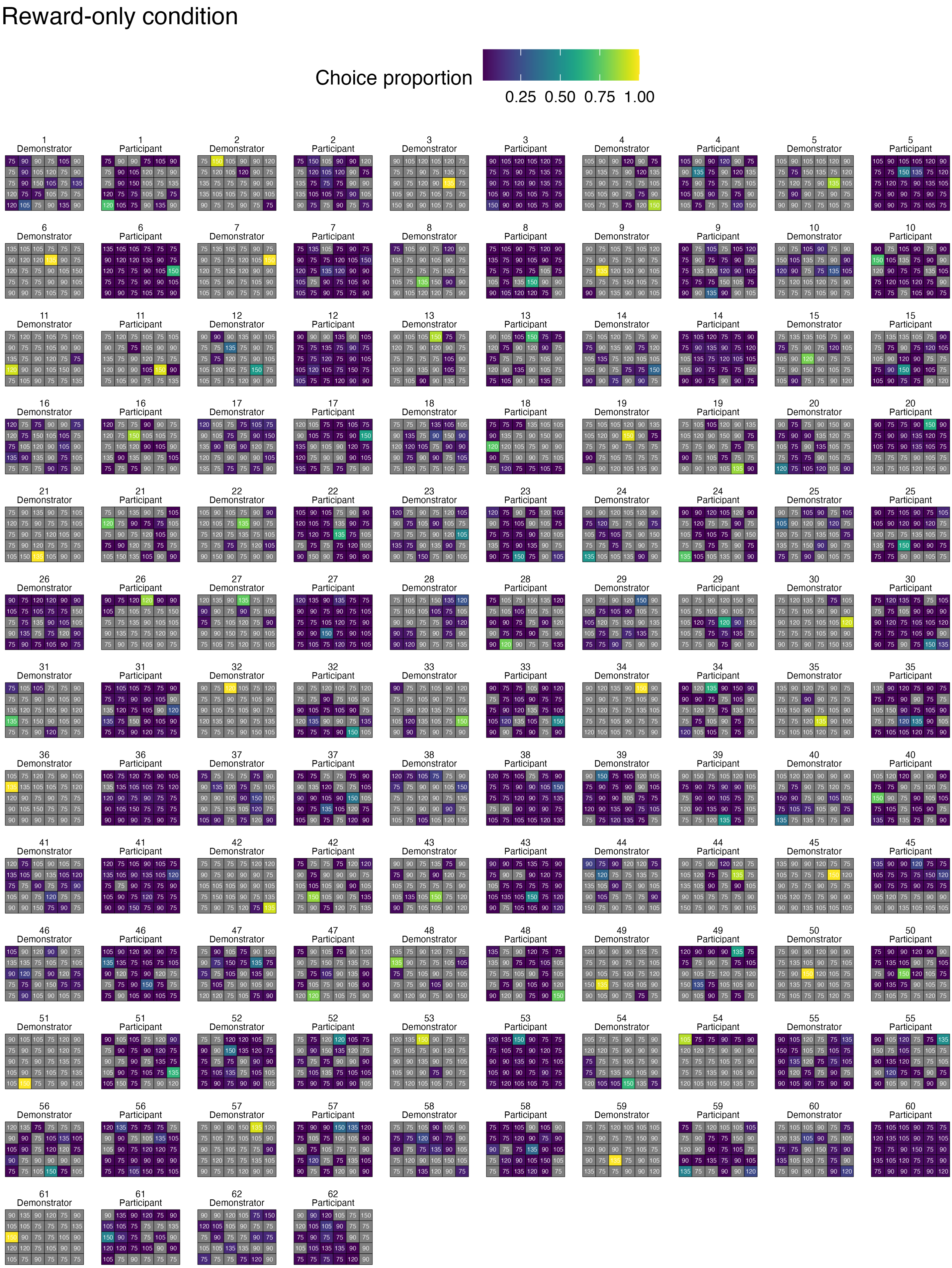

Fig. S7. Proportions of the demonstrator's choices in observational trials (left) and the participant's choices in independent trials (right) over the 30 options (Reward-only condition).

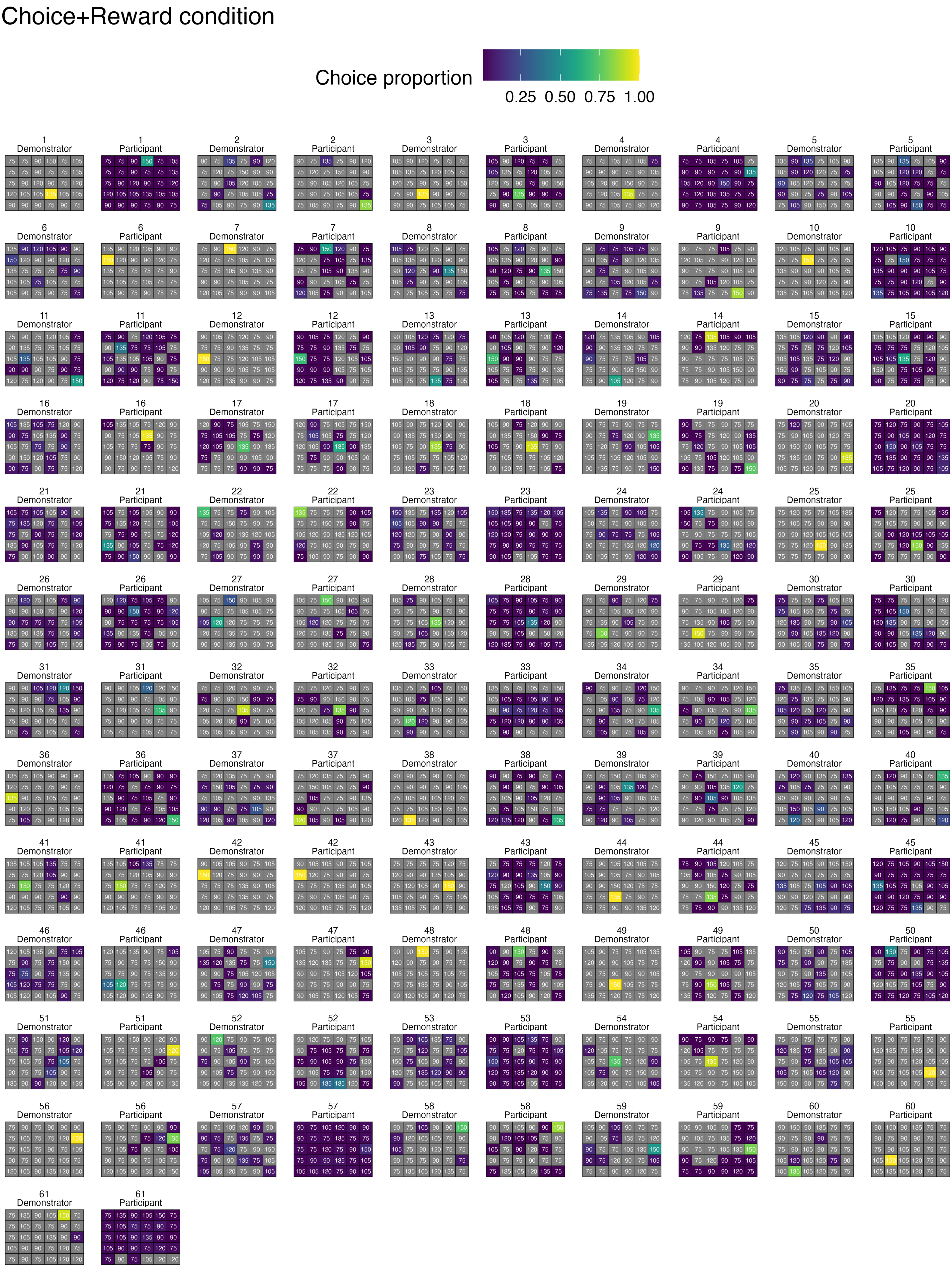

Fig. S8. Proportions of the demonstrator's choices in observational trials (left) and the participant's choices in independent trials (right) over the 30 options (Choice-plus-reward condition).

5. Exploration rates under an alternative definition

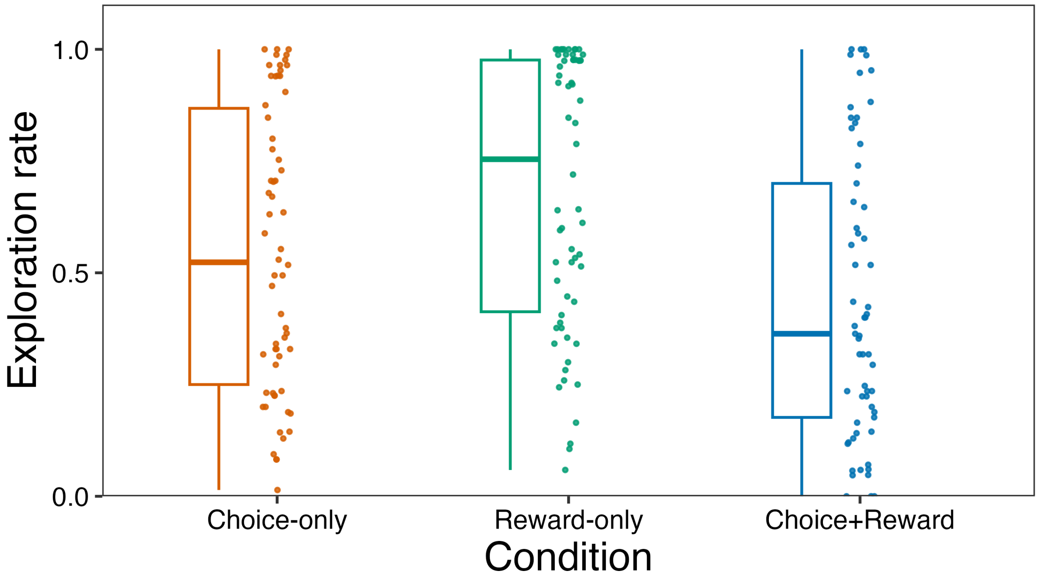

Fig. S9. Exploration rates based on the combined benchmark with the best option during observation and that directly experienced after independence. Although participants in the Choice-only and Reward-only conditions were not able to discriminate the best option during the observation period, we calculated them as if the best option were known from social information. The exploration rate here became higher than in the original definition, suggesting that quite a few participants failed to set the best option during the observation period as the default for their independent search. The exploration rate was the highest in the Reward-only condition (*Med* = 0.75), moderate in the Choice-only condition (*Med* = 0.52), and the lowest in the Choice-plus-reward condition (*Med* = 0.36). It should be noted the difference between the Choice-only and Choice-plus-reward was not statistically significant in this analysis (Wilcoxon rank-sum test: $W = 2263$, adjusted $p = .053$), but the overall pattern did not change qualitatively.

References

Sutton, R. S., & Barto, A. G. (2018). *Reinforcement Learning: An Introduction* (2nd ed.). MIT Press.

Watanabe, S. (2013). A Widely Applicable Bayesian Information Criterion. *Journal of Machine Learning Research*, *14*(1), 867–897. https://doi.org/10.5555/2567709.2502609

Wilson, R. C., & Collins, A. G. (2019). Ten simple rules for the computational modeling of behavioral data. *eLife*, *8*. https://doi.org/10.7554/eLife.49547
